# Does long‐term Bt rice planting pose risks to spider communities and their capacity to control planthoppers?

**DOI:** 10.1111/pbi.13358

**Published:** 2020-03-09

**Authors:** Zengbin Lu, Cong Dang, Fang Wang, Zhicheng Liu, Jie Chen, Yu Wang, Hongwei Yao, Qi Fang, Yufa Peng, Angharad M. R. Gatehouse, Hongxia Hua, Gongyin Ye

**Affiliations:** ^1^ State Key Laboratory of Rice Biology & Ministry of Agricultural and Rural Affairs Key Laboratory of Molecular Biology of Crop Pathogens and Insects Institute of Insect Sciences Zhejiang University Hangzhou China; ^2^ Hubei Insect Resources Utilization and Sustainable Pest Management Key Laboratory College of Plant Science and Technology Huazhong Agricultural University Wuhan China; ^3^ State Key Laboratory for Biology of Plant Diseases and Insect Pests Institute of Plant Protection Chinese Academy of Agricultural Sciences Beijing China; ^4^ School of Natural and Environmental Sciences Newcastle University Newcastle upon Tyne UK

**Keywords:** Bt rice, spider community, rice planthoppers, bio‐control services, PRC analysis

Genetically modified crops expressing insecticidal Cry proteins from *Bacillus thuringiensis* (Bt) have been grown commercially since 1996 and have successfully controlled target pests, reduced insecticide usage and increased yields. A major concern is that Bt crops may trigger outbreaks of non‐target herbivores against which they are not effective, by reducing bio‐control services provided by natural enemies, as seen with broad‐spectrum insecticides. Large‐scale and multi‐trophic field studies with Bt cotton in China have demonstrated that reduction in insecticide use associated with the growing area of this crop was related to outbreaks of the non‐target pest, the mirid bug. In contrast, Bt cotton promoted bio‐control services against aphids in landscape crops (Lu *et al.*, 2012; Li *et al.*, 2020). The long‐term effects of growing Bt rice, for controlling the lepidopteran pests (stem borers and leaf folders), on non‐target organisms and their ecological services have not previously been estimated. These data would allow the potential of Bt rice to improve integrated pest management (IPM) in rice agroecosystems to be addressed.

Spiders are the most abundant invertebrate predators and play an important role in pest control in rice ecosystems (Marc *et al.*, 1999). Over a 10‐year (2000–2004 and 2008–2012) monitoring period, 6, 022 individuals belonging to 31 spider species were collected from fields (4, 500 m^2^) growing either Cry1Ab rice (2, 950 individuals) or its near isogenic control line XS11 (3, 072 individuals) in Zhejiang Province (Zhejiang University Experimental Station, Hangzhou, China). A separate study over a shorter period (2009‐2013) collected 1, 139 individuals of spiders from fields (3, 750 m^2^) growing either Cry2Aa rice (526 individuals) or its near isogenic control line MH63 (613 individuals) in Hubei Province (Yuanzhongchang Farmland, Xiaogan, China). Principal response curve (PRC) analyses showed that no significant differences were detected in the spider community between Bt and non‐Bt rice fields (Figure 1a). The rice genotype accounted for only a small proportion of the variance in the spider community between Bt rice and its control (Cry1Ab rice: 5.9 %; Cry2Aa rice: 8.8 %), while the sampling times accounted for a greater proportion of the total variance (Cry1Ab rice: 61.8%; Cry2Aa rice: 56.6%). The spider species, *Tetragnatha maxillosa*, *Oedothorax insecticeps*, *Lycosa pseudoamulata*, *Theridonn octomacutatum* and *Pirata subparaticus,* differed in abundance between Cry1Ab rice and its control (Figure 1a‐1). Additionally, *Tetragnatha* sp., *P. subpiraticus* and *O. insecticeps* strongly affected the response curve in Cry2Aa rice (Figure 1a‐1). However, as expected, the spider community in Bt and non‐Bt rice fields was significantly reduced by insecticide sprays compared with that of untreated non‐Bt rice in both years (Figure 1b), primarily due to the reduction in three spider taxa (*P. subparaticus, T. maxillosa* and *O. insecticeps*). Importantly, spider communities did not differ between unsprayed Bt and unsprayed non‐Bt rice (Figure 1b). These results suggest that Bt rice has no long‐term impacts on the structure of the spider community, while chemical insecticides exhibit negative impacts.

**Figure 1:**
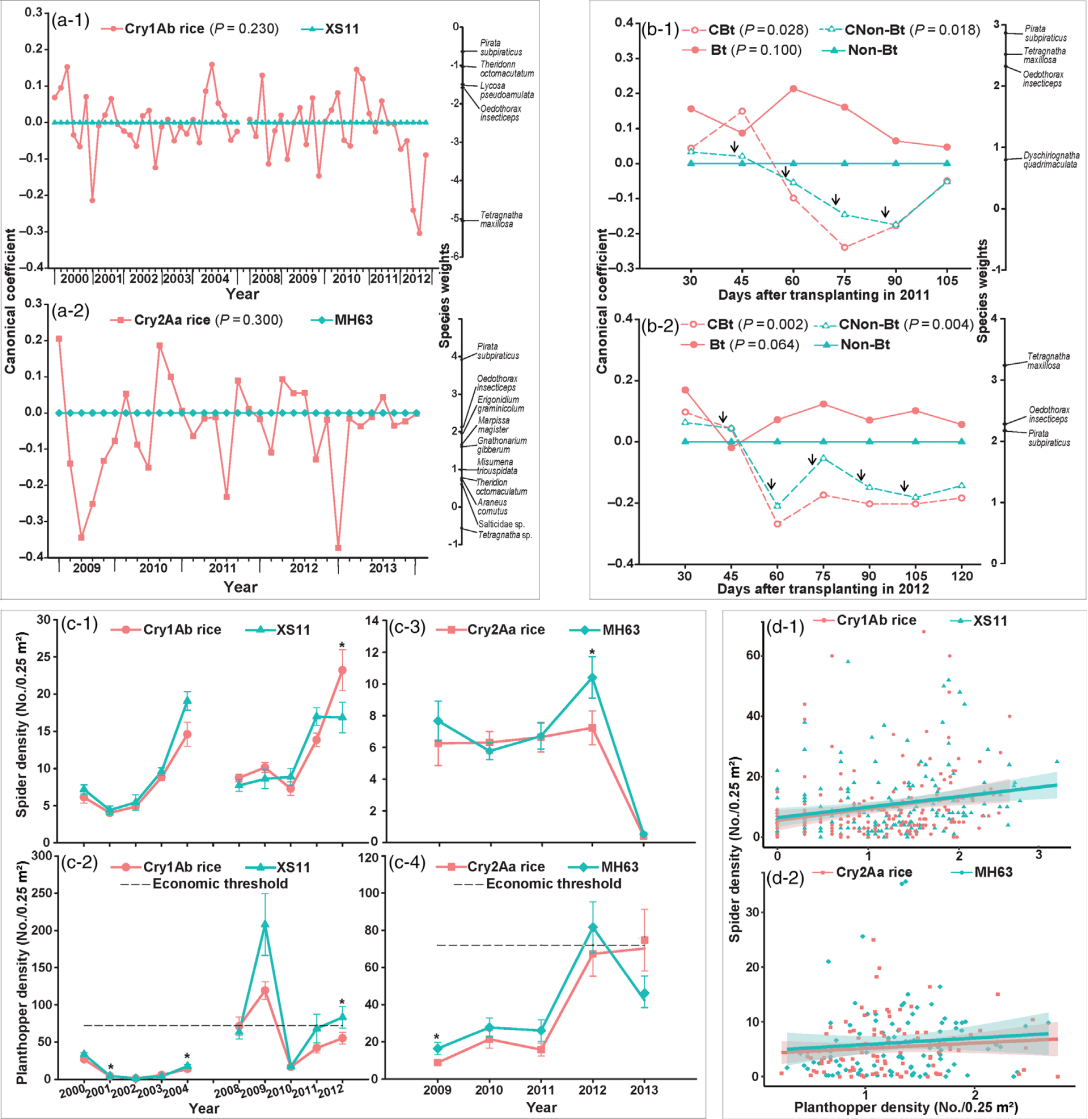
(a) PRC and species weights of the spider community collected in Bt and unsprayed non‐Bt rice fields. (a‐1) Cry1Ab rice and its parental line XS11; a‐2, Cry2Aa rice and its parental line MH63. Non‐Bt plots were used as the reference. Taxa with higher or lower species weights (>0.5 or < −0.5) strongly influenced the shape of the treatment response curves. For clarity, taxa with species weights between −0.5 and 0.5 are not shown. (b) PRC and species weights of the spider community collected in rice fields in Zhejiang Province with the following 4 treatments in 2011 (b‐1) and 2012 (b‐2): Bt rice sprayed with chemical pesticides (CBt), sprayed non‐Bt rice (CNon‐Bt), unsprayed Bt rice (Bt) and unsprayed non‐Bt rice (Non‐Bt) fields. Arrows indicate that insecticide was applied (always three days before the sampling date). The rice lines were Cry1Ab rice and XS11. Chemical pesticides were methamidophos and bisultap. For clarity, taxa with species weights between −0.5 and 0.5 are not shown. (c) Seasonal densities of spiders and rice planthoppers in Bt and unsprayed non‐Bt rice fields. (c‐1) spider density in Cry1Ab rice and XS11 field, *F*_rice line_ = 0.37, *df* = 1, *P*_rice line_ = 0.560; *F*_year_ = 34.84, *df* = 9, *P*_year_ < 0.001; *F*_rice line*year_ = 1.75, *df* = 9, *P*_rice line*year_ = 0.094; (c‐2) planthopper density in Cry1Ab rice and XS11 field, *F*_rice line_ = 8.71, *df* = 1, *P*_rice line_ = 0.018; *F*_year_ = 305.87, *df* = 9, *P*_year_ < 0.001; *F*_rice line*year_ = 1.93, *df* = 9, *P*_rice line*year_ = 0.061; (c‐3) spider density in Cry2Aa rice and MH63 field, *F*_rice line_ = 2.38, *df* = 1, *P*_rice line_ = 0.198; *F*_year_ = 116.29, *df* = 4, *P*_year_ < 0.001; *F*_rice line*year_ = 1.13, *df* = 4, *P*_rice line*year_ = 0.378; (c‐4) planthopper density in Cry2Aa rice and MH63 field, *F*_rice line_ = 2.16, *df* = 1, *P*_rice line_ = 0.215; *F*_year_ = 24.74, *df* = 4, *P*_year_ < 0.001; *F*_rice line*year_ = 2.20, *df* = 4, *P*_rice line*year_ = 0.115. Asterisk * indicates significant difference between Bt and non‐Bt rice fields in the same year, *P* < 0.05. The black dashed lines indicate the economic threshold of planthoppers (72 individuals per 0.25 m^2^). Error bars represent standard error (SE). (d) Relationships between spider density (*y*) and rice planthopper density (*x*, log_10_(*n* + 1) transformed) in Bt rice and unsprayed non‐Bt rice fields. (d‐1) Cry1Ab rice: *y* = 3.84*x* + 5.56, *r* = 0.22, *P* = 0.001; XS11: *y* = 3.51*x* + 6.45, *r* = 0.23, *P* = 0.001; two slopes compared: *F* = 0.04, *P* = 0.844. d‐2, Cry2Aa rice: *y* = 0.95*x* + 4.18, *r* = 0.11, *P* = 0.155; MH63: *y* = 1.15*x* + 4.69, *r* = 0.09, *P* = 0.188; two slopes compared: *F* = 0.02, *P* = 0.899. Predictions with linear models are plotted along with upper and lower 95% confidence intervals.

Repeated‐measures ANOVA also revealed no significant difference in the seasonal densities of spiders between Bt and non‐Bt rice fields (Figure 1c‐1 and c‐3). However, the abundance of rice planthoppers (including *Nilaparvata lugens*, *Sogatella furcifera* and *Laodelphax striatellus*), the most important non‐target sap‐sucking herbivores in Bt rice fields, was significantly reduced by Cry1Ab rice but not affected by Cry2Aa rice (Figure 1c‐2 and c‐4). Rice planthopper densities in Cry1Ab rice fields (except in 2009) and Cry2Aa rice fields (all sampling years) remained below the economic threshold of 72 individuals per 0.25 m^2^ (Li *et al.*, 2006). The two Bt rice lines did not trigger rice planthopper outbreaks, since they retained the bio‐control services of spiders. Positive correlation between the abundance of spiders and planthoppers in both Bt and non‐Bt fields without insecticides over the long‐term survey could strongly support it (Figure 1d‐1 and d‐2). However, this correlation was relatively weak (*P* < 0.200) in Cry2Aa rice fields.

Chemical insecticides have been extensively used to control rice planthoppers throughout Asia, including China. However, the misuse of these insecticides has reduced the abundance of natural enemies and disrupted predator–prey relationships and the food web structure, thus favouring population increases of rice planthoppers (Heong and Schoenly, 1998). Transgenic Bt rice lines have been developed to protect crops against lepidopteran pests while mitigating these non‐target negative effects of synthetic insecticides. Previous field tests, albeit of limited duration (≤3 years), have shown that Bt rice had no negative effects on abundance of spiders (Dang *et al.*, 2017). Several non‐target herbivore pests, such as whiteflies, mirid bugs and aphids, increased in number in Bt cotton and Bt maize fields, causing significant economic loss; these were intrinsically linked with reduced insecticide use (Catarino *et al.*, 2015). In contrast, rice planthopper and thrip densities were lower in Bt rice fields, and varied among different Bt rice lines (Dang *et al.*, 2017). The different performance of non‐target herbivores in Bt rice might result from bio‐control services of natural enemies, as shown in the present study. The unintended effects as a consequence of genetic manipulation within the rice genome (Wang *et al.*, 2015) and interspecific interactions among herbivores (Wang *et al.*, 2018) could also account for this difference.

In conclusion, our long‐term study shows that growing two different Bt rice lines did not induce planthoppers outbreak. Importantly, we also showed that these Bt rice lines have no negative effects on the spider sub‐community or on their ecological services, in contrast to the use of broad‐spectrum insecticides. These results clearly indicate that Bt rice is compatible with IPM (Romeis *et al.*, 2019), even in the long term.

## Conflict of interest

The authors declare no conflict of interest.

## Author contributions

G.Y.Y., H.X.H., A.M.R.G., Y.F.P., Z.B.L. and C.D. contributed to study design and data analysis; Z.B.L. and C.D. equally contributed to overall study, arthropod identification and data analysis; Z.C.L., F.W., H.W.Y., Q.F., J.C. and Y.W. contributed to field sampling and some arthropod identification; and Z.B.L., C.D., A.M.R.G. and G.Y.Y. wrote the manuscript. All authors read and approved the paper.
